# Extracellular vesicle as biomarkers in NSAID‐exacerbated respiratory disease

**DOI:** 10.1002/clt2.70028

**Published:** 2025-02-13

**Authors:** Isaac Kirubakaran Sundar

**Affiliations:** ^1^ Division of Pulmonary, Critical Care, and Sleep Medicine Department of Internal Medicine University of Kansas Medical Center Kansas City Kansas USA

We found the recent Allergy article titled “Extracellular vesicle miRNAs drive aberrant macrophage responses in NSAID‐exacerbated respiratory disease” fascinating. We commend the authors for their exciting research into the role of extracellular vesicle (EV) miRNAs in mediating aberrant macrophage responses in NSAID‐exacerbated respiratory disease (N‐ERD).^1^ The authors evaluated EVs from sputum and conditioned medium of the nasal polyp or turbinate tissues from patients with chronic rhinosinusitis with nasal polyps (CRSwNP), N‐ERD, and healthy controls.^1^ Small RNA sequencing revealed intriguing and contrasting miRNA profiles in sputum EVs from N‐ERD patients compared to previous reports on BAL fluid EVs from asthmatics,^2^ and nasal tissue from CRSwNP patients.^3^ Specifically, let‐7 family miRNAs were upregulated while miR‐155 was downregulated in N‐ERD sputum EVs.^1^ These distinct miRNA signatures suggest the source of EVs dictates the enrichment of miRNAs in healthy versus N‐ERD samples. Additionally, in vitro experiments supported a role for let‐7 family miRNAs in promoting M2 macrophage polarization, which limits cytokine responses triggered by EVs in monocyte‐derived macrophages (MDM). This implicates aberrant EV miRNA profiles contributing to N‐ERD pathogenesis.

The results obtained from small RNA sequencing analysis are interesting, but the authors have not verified their findings through alternative methods or a replication cohort of samples. They have not provided sufficient details about the total EV concentration and EV protein abundance across the three groups (healthy, CRSwNP, and N‐ERD) and the sample types analyzed (sputum vs. turbinate tissue/nasal polyp tissue). It is also unclear whether the EV miRNAs identified match previous findings in similar sample types from asthma and IPF.^4^, ^5^ Moreover, the use of pooled samples and a relatively small sample size for the RNA‐sequencing and interpretation is surprising. To support the RNA‐seq data presented here, direct validation of the miRNA signature and associated mRNA targets in the N‐ERD groups is necessary. We have pointed out the strengths and weaknesses of this study below. Overall, we advise caution in interpreting these findings until they are validated in a larger sample cohort by the authors and readers.

This study sheds light on the pathogenesis of N‐ERD, which is a severe form of asthma. The study analyzed the RNA‐seq data from MDM treated with sputum EVs from healthy, CRSwNP, and N‐ERD patients, as well as small RNA‐seq of sputum and tissue culture supernatant EVs from healthy turbinate or nasal polyp tissue (N‐ERD). Although the authors performed a comprehensive analysis of EV miRNA profiling and in vitro experiments using MDM, the identified EV‐specific miRNAs (upregulated miR‐125a and let‐7 family) enriched in N‐ERD sputum EVs induced M2 polarization and enhanced proinflammatory and tissue remodeling functions. This provides initial mechanistic insights into N‐ERD pathobiology that warrant further exploration and validation. However, the study did not discuss known EV miRNA findings from asthma studies nor integrate the RNA‐seq and small RNA‐seq data from MDM experiments to directly relate target genes and associated miRNAs to cell type‐specific roles in N‐ERD versus healthy controls. Overall, while this study made important contributions, integrating past literature and transcriptomic datasets within the study could provide deeper novel insights into miRNA‐mediated mechanisms in N‐ERD.

Asthma is a complex, heterogeneous disease with diverse phenotypes and endotypes, which makes it difficult to diagnose and treat.^6^ The “one‐airway‐one‐disease” theory suggests that the upper and lower airways share similar characteristics, but the mechanisms behind asthma are diverse and sometimes overlap.^7^ Recently, researchers have found that studying EVs through liquid biopsy can provide new insights into how they signal and regulate lung health and disease.^8^ Identifying new EV biomarkers that can distinguish asthma phenotypes and endotypes^9^ is necessary because current biomarkers are not sensitive and specific.

However, this study has some limitations. The authors used RNA‐seq and small RNA‐seq to identify genes and miRNAs in MDM treated with sputum EVs from healthy controls and patients with N‐ERD (*n* = 2). The exploratory studies used pooled sputum EVs from N‐ERD patients (*n* = 4) and healthy controls (*n* = 10), as well as nasal tissue culture supernatant EVs from healthy controls (*n* = 1) and N‐ERD patients (*n* = 4), with very small sample sizes. This raises concerns about whether these findings can be reproduced in a larger validation cohort that compares healthy controls, CRSwNP, and N‐ERD patients. This study did not provide detailed clinical characteristics of the healthy controls, N‐ERD patients, and CRSwNP patients, and relevant variables were omitted, including medication use, comorbidities, and disease severity, which may affect the EV miRNA signatures identified. The supporting experimental evidence was obtained using cell culture models with pooled EV samples and MDM from patients. More studies using relevant in vivo models could strengthen these findings, provide mechanistic insights, and identify potential therapeutic targets specific to N‐ERD and CRSwNP.

The authors of this study did not functionally validate their findings beyond discussing the potential signaling pathways that may have contributed to the aberrant activation of macrophages and the mediator response observed in N‐ERD. Therefore, the findings reported in this study are specific to N‐ERD and should be interpreted with caution as they may not apply to other forms of asthma or respiratory diseases. Phenotypic differences due to age and sex/gender also likely play a role. To strengthen the exploratory analysis of small RNAs in EVs, their associated target genes, and their functional contributions to the pathobiology of N‐ERD and CRSwNP, it would be worthwhile for the authors to validate these findings using easily accessible samples from healthy controls, N‐ERD, and CRSwNP patients with detailed clinical characterization. Future research should focus on using easily accessible liquid biopsy samples from N‐ERD and CRSwNP to characterize unique EV miRNA as biomarkers for asthma phenotypes/endotypes. This would enable the development of new strategies to target EV miRNAs to modulate aberrant macrophage activation and associate inflammatory signaling that plays a crucial role in chronic airway disease pathobiology.

## AUTHOR CONTRIBUTIONS

**Isaac Kirubakaran Sundar**: Writing—original draft; writing—review & editing.

## CONFLICT OF INTEREST STATEMENT

The author declares no conflicts of interest.

## FUNDING INFORMATION

National Heart, Lung, and Blood Institute, R01HL142543; National Institute of General Medical Sciences, K‐INBRE P20 GM103418; University of Kansas Medical Center, School of Medicine, Internal Medicine Start‐Up Funds.

## Data Availability

Data sharing not applicable to this article as no datasets were generated or analyzed during the current study.
